# Hemispheric lateralization of Tau associations with visual and verbal memory in Alzheimer’s disease

**DOI:** 10.1007/s00259-026-07898-z

**Published:** 2026-06-05

**Authors:** Jaime Fernández Arias, Etienne Aumont, Joseph Therriault, Lydia Trudel, Nina Margherita-Poltronetti, Emilie Thomas, Gleb Bezgin, Stijn Servaes, Yi-Ting Wang, Nesrine Rahmouni, Arthur Macedo, Kely Quispialaya, Seyyed Ali Hosseini, Brandon Hall, Peter Kunach, Wan Lu Jia, Tevy Chan, Yansheng Zheng, Marcel Seungsu Woo, Sulantha S. Mathotaarachchi, Paolo Vitali, Tharick Pascoal, Yasser Iturria-Medina, Maxime Montembeault, Pedro Rosa-Neto

**Affiliations:** 1 Translational Neuroimaging Laboratory, Montreal Neurological Institute, Montreal, H3A 2B4 Canada; 2 McGill University Research Centre for Studies in Aging, Verdun, H4H 1R3 Canada; 3 Faculty of Medicine, McGill University, Montreal, H3G 2M1 Canada; 4 Université du Quebec á Montréal, Montreal, H2L 2C4 Canada; 5 Douglas Mental Health University Institute, Verdun, H4H 1R3 Canada; 6 Department of Neurology and Psychiatry, University of Pittsburgh School of Medicine, Pittsburgh, 15213 USA; 7 Diamond Lab, University of Texas Southwestern, Dallas, 75390 USA; 8 Department of Neurology, University Medical Center Hamburg Eppendorf, 20151 Hamburg, Germany; 9 Department of Neurology and Neurosurgery, Montreal Neurological Institute, Montreal, H3A 2B4 Canada

**Keywords:** Memory, Tau PET, Lateralization, Laterality index, Alzheimer’s disease

## Abstract

**Purpose:**

Alzheimer’s disease affects the brain in complex ways, and the location of tau tangles may influence specific types of memory problems. In this study, we examined how tau accumulation relates to verbal and visual memory performance, and whether these associations show a preference for one hemisphere of the brain over the other.

**Methods:**

We administered the Rey Auditory Verbal Learning Test (RAVLT) and its nonverbal analog, the Aggie Figures Learning Test (AFLT), to 132 cognitively unimpaired elderly participants and 44 cognitively impaired, amyloid-positive individuals from the Translational Biomarkers in Aging and Dementia cohort. All participants underwent [18 F]MK6240 tau PET, [18 F]AZD4694 amyloid PET, and structural MRI scans. We analyzed the data using region-of-interest and voxel-wise regression models, as well as correlation analyses.

**Results:**

Voxel-wise analyses showed that higher tau load in the right hemisphere was associated with worse visual memory, confirming a lateralized relationship. Left-hemisphere tau associations with verbal memory were observed at higher t-values. In regression models including both memory scores, visual memory remained significantly associated with tau on the right hemisphere (*β*_*AFLT~Rtau*_=-0.22, *p*=0.001; *β*_*RAVLT~Rtau*_=-0.1, *p*=0.13), whereas both visual and verbal memory remained significant on the left hemisphere (*β*_*AFLT~Ltau*_=-0.16, *p*=0.03; *β*_*RAVLT~Ltau*_=-0.14, *p*=0.04). These patterns were also reflected in Braak regions, except for Braak I, where only left tau–verbal and right tau–visual associations remained significant.

**Conclusion:**

Our findings support lateralized associations between tau accumulation and memory deficits, with visual memory linked to right-hemisphere tau and verbal memory to left-hemisphere tau. This pattern is consistent with lateralization of memory functions observed in other neurological conditions and highlights the importance of considering hemisphere-specific tau pathology in Alzheimer’s disease.

**Supplementary Information:**

The online version contains supplementary material available at https://doi.org/10.1007/s00259-026-07898-z.

## Introduction

Alzheimer’s disease (AD) is characterized by the accumulation of amyloid and tau aggregates [1]. The anatomical distribution of tau aggregates has been associated with atypical syndromes and typical AD symptoms. In particular, anterograde memory deficits are related to tau load in the medial temporal lobe (MTL) and other cortical regions that are part of memory networks [2, 3]. However, this relationship is difficult to disentangle because both tau accumulation and memory decline progress concurrently across cortical regions [4]. 

Verbal and non-verbal memory are commonly distinguished within episodic memory research and appear to rely on partially distinct neural systems [5–21]. In practice, this distinction is often based on the type of material presented during the encoding phase rather than on strict formal definitions. Verbal memory refers to the encoding and retrieval of linguistic information and primarily engages a left-lateralized frontotemporal network that includes the inferior frontal gyrus [10], and temporal structures such as the hippocampus and the parahippocampus [7, 22, 23]. Visual memory, in contrast, refers to the encoding and retrieval of non-linguistic, spatial, or figural material, which tends to preferentially recruit right-hemisphere regions, including the right hippocampus, the fusiform gyrus, and the occipito-parietal cortices [7, 22–24]. 

Hemispheric specialization of these memory systems has been consistently implied across methodologies. Neuropsychological studies of patients with unilateral temporal lobe damage show material-specific impairments: left temporal lesions lead to verbal memory deficits, while right temporal lesions result in visual or spatial memory impairments [5, 6, 14, 16–18, 25]. Functional MRI studies further reveal that the left inferior prefrontal cortex (IPC) and left dorsolateral prefrontal cortex (DLPFC) are preferentially activated during encoding of verbal material, whereas the right DLPFC is engaged during encoding of non-verbal material [21]. Within the temporal lobe, early fMRI studies have suggested a similar left–right specialization in structures of the medial temporal lobe and the fusiform gyrus, although other regions (e.g., the parahippocampus) show less consistent patterns [21, 26]. The right perirhinal cortex (PRC) has also been specifically implicated in non-verbal encoding tasks [27]. Other studies, however, reveal a more complex picture [28, 29]. In both verbal and non-verbal cases, posterior medial parietal cortices such as the retrosplenial (RSC) and posterior cingulate (PCC) are also key nodes of the episodic memory network [24]. 

From a theoretical perspective, this material-specific lateralization suggests that memory functions are supported by distinct, though interconnected, hemispheric networks that integrate domain-specific cortical regions with the hippocampal formation [30]. In Alzheimer’s disease, tau pathology propagates along functional and structural networks that often mirror this normative organization [31]. Consequently, tau accumulation within left-lateralized verbal memory circuits could preferentially disrupt verbal learning and recall, while tau deposition in right-lateralized visual networks could more strongly impact visual memory performance. If this is the case, there are potential implications for both clinical and research practice. For example, PET studies in the field of Alzheimer’s disease frequently use composite memory scores that combine several memory measures together, but if underperformance in various tests signals variability in underlying neuropathology, composite memory scores do not provide a granular understanding of these subtleties. Furthermore, testing these associations provides an opportunity to determine whether tau-related neurodegeneration respects the functional architecture of human memory systems, or whether tau–memory correlations simply reflect overall disease burden.

Following this paradigm, if the relationship between tau load and memory dysfunction were merely parallel, similar correlations between tangles and performance on verbal or visual memory tests would be expected across hemispheres. In contrast, hemispheric differences would support a causal, functionally specific association between tangle load and domain-specific memory deficits. Prior studies examining this question have yielded inconsistent results [2, 3, 32–35], likely due to methodological limitations such as non-equivalent memory tests and the use of first-generation tau tracers [36]. 

To address these issues, we examined the lateralization of associations between tau pathology and verbal or visual memory using [^18^F]MK6240, a second-generation tau PET tracer with high specificity for tau fibrils [37]. We employed two analogous recall tests that minimize procedural differences between verbal and non-verbal conditions: the Rey Auditory Verbal Learning Test (RAVLT) [38] and the Aggie Figures Learning Test (AFLT) [39]. 

Since the laterality of brain lesions is associated with domain-specific memory impairments [8], we hypothesized that verbal performance would display a stronger relationship with tau tangle load in the left hemisphere, while nonverbal performance would show a stronger association with tau in the right hemisphere.

Moreover, we predicted that hemispheric asymmetry in tau-related measures, quantified using laterality indices (LI) [40], would be differentially related to recall scores in the specified directions. Laterality indices have been used to characterize hemispheric asymmetries in Alzheimer’s disease biomarkers, including studies examining asymmetric tau deposition or the relationship between amyloid and tau asymmetry [41, 42]. In the present study, we extend this framework by computing multiple LIs, including indices based on hemispheric tau burden as well as indices derived from the strength of tau–memory associations across hemispheres.

## Materials and methods

### Study samples

We assessed 176 individuals from the Translational Biomarkers of Aging and Dementia (TRIAD) cohort: [43] 132 cognitively unimpaired (CU) older adults and 44 cognitively impaired (CI), amyloid-β positive individuals. Among the CI participants, 27 had mild cognitive impairment (MCI) and 17 had a diagnosis of Alzheimer’s disease. All participants underwent amyloid-PET with [^18^F]AZD4694 and tau-PET with [^18^F]MK6240. Moreover, they received a neurological examination by a physician, a review of their medical history and a neuropsychological assessment that included tests for visual and verbal memory. CU individuals had no objective cognitive impairment and a Clinical Dementia Rating (CDR) score of 0. MCI and AD individuals were diagnosed based on the NIA-AA criteria for MCI due to Alzheimer’s disease [44] and for probable Alzheimer’s disease dementia [45], respectively, by a multidisciplinary team of neurologists, neuropsychologists and nurses. Exclusion criteria included medical conditions not under a stable medication regimen, active substance abuse, recent head trauma or major surgery, or MRI/PET safety contraindications. The time lapse between PET scans, MRI scans and neuropsychological assessments was less than a year in the vast majority of cases (the average number of days between appointments ranged from 6 to 32 days). There were two individuals whose neuropsychological tests were done with a difference of over a year. Additionally, there were two individuals whose difference between the date of the neuropsychological assessment and the tau PET scan was also over a year. For a full overview of the results, see Supp. Figure 1.

### Memory testing

Verbal memory was assessed using delayed recall of the Rey Auditory Verbal Learning Test (RAVLT) [38]. The RAVLT consists of a list of 15 words (List A) that is presented in auditory format at a rate of one word per second over 5 *learning* trials. After each time the list is presented, participants are asked to recall the words that they remember. After the 5th trial, another list of 15 words (List B) is presented and participants are asked to call out the words from List B. Immediately afterwards, they are asked to recall the words that they remember from List A. This 6th trial is sometimes referred to as the interference trial. Finally, after a delay of 20–25 min, participants are asked to recall the words that they remember from List A. This is the delayed recall trial, which we used in this manuscript.

Visual memory was tested using the Aggie Figures Learning Test (AFLT) [39], which is considered a visual analog of the RAVLT. In this case, however, hard-to-verbalize figures replace the words, presentation time per figure is increased to 3 s, and participants are asked to draw the figures that they recall after every trial.

For both tests, raw scores were z-transformed using mean and standard deviation (s.d.) values from the whole sample.

### Grey matter volume

We included an estimate of grey matter volume as a potential confounder. T1-weighted MRIs were acquired via an MPRAGE sequence at the Montreal Neurological Institute on a 3 T Siemens Magnetom using a standard head coil. Non-uniformity and field-distortion corrections were conducted using Advanced Normalization Tools (ANTS). Grey matter volume indicators were extracted from FreeSurfer [46], version 5.0.9, using the recon-all command. Since a raw indicator of total gray volume may be misleading, as the differences in head sizes across sexes may act as a confounder, we applied a correction that consisted of residualizing the total gray matter volume on the total intracranial volume, as previously described [47]. 

### PET scans

[^18^F]AZD4694 PET and [^18^F]MK6240 PET scans were conducted with a brain-dedicated Siemens High Resolution Research Tomograph (HRRT). [^18^F]AZD4694 PET images were acquired 40–70 min after bolus injection and reconstructed with the ordered-subset expectation maximization algorithm (OSEM) on a four-dimensional volume with three frames (6 × 300 s), as previously described [48]. [^18^F]MK6240 PET images were acquired at 90–110 min after bolus radiotracer injection and reconstructed with the OSEM algorithm on a four-dimensional volume with four frames (4 × 300 s). A 6-min transmission scan with a rotating 137Cs point source was performed after each PET acquisition for attenuation correction. Corrections to PET images were applied for decay, motion, dead time, random, and scattered coincidences. T1-weighted MRIs were processed using an in-house pipeline after the corrections mentioned in the previous section. PET images were automatically registered to the T1-weighted image space, and the T1-weighted images were linearly and non-linearly registered to the Alzheimer’s Disease Neuroimaging Initiative (ADNI) reference space. [^18^F]MK6240 images were meninges-stripped in native space before they were transformed and blurred to minimize interference from meningeal spillover, as previously described [49]. The whole cerebellum gray matter was used as the reference region to calculate [^18^F]AZD4694 standardized uptake value ratio (SUVR) maps, as this region has long been found to provide good contrast with regions where there is an increase in amyloid PET signal in AD individuals [50]. The inferior cerebellar gray was used as the reference region to generate [^18^F]MK6240 SUVR maps, since it appears to optimize the ratio in cross-sectional analyses compared to other methods such as the full cerebellar gray [51]. Spatial smoothing allowed the PET images to achieve an 8-mm full-width at half-maximum resolution.

Amyloid-β SUVR was estimated for each participant by averaging the SUVR from a region of interest (ROI) that encompassed the precuneus, prefrontal, orbitofrontal, parietal, temporal, and cingulate cortices [52]. Amyloid-β positivity was defined as a global [^18^F]AZD4694 SUVR greater than 1.55 [48]. Temporal meta-ROI SUVR (tau PET) was calculated by averaging [^18^F]MK6240 SUVR values from entorhinal, parahippocampal, amygdala, fusiform, inferior, and middle temporal cortices. Tau positivity was defined as two s.d. above the average SUVR of cognitively unimpaired young (< 26 years old) individuals, as previously described [53], which yielded a threshold of 1.19.

### PET-Braak staging

A full description of the method for PET-based Braak staging can be found elsewhere [49]. Braak stages were based on anatomical brain regions suggested by Braak [31]. PET-Braak-defined stages included: transentorhinal cortex (PET-Braak I), entorhinal cortex and hippocampus (PET-Braak II), inferior temporal neocortex (PET-Braak III), association cortices (PET-Braak IV and V) and primary sensory cortices (PET-Braak VI) [49]. Participants were assigned PET-Braak stages based on the latest stage where tau PET abnormality was identified by an automatic pipeline [49]. Segregation into higher PET-Braak stages was determined only if the cutoff in lower stages had also been reached. The threshold for tau PET abnormality across PET-Braak regions was defined as 2.5 standard deviations (s.d.) higher than the mean SUVR of CU young adults, a previously set standard [49]. Consistent with the correspondence between the probability of Alzheimer’s disease dementia diagnosis and Braak stages [54], 76% of our PET-Braak I and II participants were CU elderly and only 6–7% of them had a diagnosis of probable Alzheimer’s disease. By contrast, 72% of participants assigned PET-Braak stages V or VI had such a diagnosis.

### Laterality indexes

We calculated three different types of lateralization indices (LIs) to address two main objectives. First, we used the LI to determine whether associations between verbal or visual memory scores and tau were stronger in the left or right hemisphere. For this purpose, we derived LIs from the t-values obtained in regression models assessing the relationship between memory scores and tau SUVR in the left and right temporal meta-ROIs (see Statistical Analyses). This measure, referred to as LI-tval, was calculated separately for verbal and visual memory. Following previous work [3], LI-tval was computed as:

$$\:(T\_left-\:T\_right)/\:(T\_left+\:T\_right\:)$$

T_left and T_right represent the t-values for the left and right hemispheres, respectively. Positive LI values indicate stronger left-hemisphere associations, while negative values indicate stronger right-hemisphere associations. In a complementary approach, we calculated LIs based on the number of voxels that remained significant at varying t-statistic thresholds for each hemisphere in the temporal meta-ROI (see voxel-wise analyses). This approach produced a range of LIs reflecting the spatial extent of memory-tau associations across hemispheres as their statistical strength increased. We referred to this LI as LI-vox. The concept had been previously applied in fMRI studies [55]. At each t-statistic threshold, the formula for the LI-vox was the following:

$$\:(N\_right-\:N\_left\:)/\:(N\_right+\:N\_left\:)$$

Where N_right is the number of significant voxels in the right hemisphere and N_left is the number of significant voxels in the left hemisphere.

Finally, we calculated a lateralization index to quantify hemispheric asymmetry in tau accumulation, which we hypothesized would differentially relate to verbal and visual memory. This measure is referred to as LI-tau. Following recent work [56], LI-tau was calculated using tau SUVRs from the left and right temporal meta-ROIs:

$$\begin{array}{c}(Left\:metaROI\:SUVR-Right\:metaROI\:SUVR){/}\\(Left\:metaROI\:SUVR+Right\:metaROI\:SUVR)\end{array}$$

Where Left metaROI SUVR is the tau SUVR in the left temporal meta-ROI and Right metaROI SUVR is the tau SUVR in the right temporal meta-ROI. LI-tau values range from − 1 to + 1, where a positive value indicates a left-hemisphere bias and a negative value indicates a right-hemisphere bias. A value of −1 represents complete right lateralization, while a value of + 1 represents complete left lateralization. In the context of MRI research, values below − 0.1 are considered right-lateralized, and values above 0.1 are considered left-lateralized [57]. 

### Voxel-wise analyses

To visually inspect the association between memory scores and tau PET, voxel-wise analyses were implemented in the whole sample. We used VoxelStats [58], with either RAVLT delayed recall scores or AFLT delayed recall scores as dependent variables and [^18^F]MK6240 SUVR as independent variable. All models were corrected for amyloid-β SUVR, grey matter volume, age, years of education, sex, APOE genotype, and handedness. The same variables were entered in additional voxel-wise analyses restricted to the temporal meta-ROI to calculate the LI-vox.

### Statistical analyses

Statistical analyses were performed in R v4.3.0. Regression models were conducted to assess the independent association between tau PET and memory scores by hemisphere and modality, respectively. Right or left tau SUVR in the temporal meta-ROI were entered as independent variables, and cognitive scores as dependent variables, with amyloid-β SUVR, grey matter volume, age, years of education, sex, APOE genotype, and handedness as covariates. We refer to these models as “simple models”. Simple models were used to calculate the LI-tval.

To further assess differences across modalities, we conducted regression analyses with verbal or visual memory scores as dependent variables, right or left tau temporal meta-ROI SUVR as independent variables, and the remaining memory scores in addition to the variables mentioned above as covariates. We refer to models including both memory tests as “combined models”. In follow-up analyses, we repeated the latter procedure across PET-Braak stages. Variance inflation factor (VIF) and tolerance values were calculated for all models to test for the presence of collinearity.

Lastly, we ran partial correlation analyses to test the relationship between LI-tau and memory scores. We conducted follow-up analyses to evaluate whether partial correlations were linked to cognitive impairment, significant tau load across PET-Braak stages, or the temporal meta-ROI. These partial correlations were conducted in cognitively unimpaired and cognitively impaired individuals, as well as participants segregated by their tau status. For these analyses, we used the *ppcor* package of R [59]. 

## Results

Demographic, clinical, and biomarker data are summarized by diagnostic category in Table 1 and by tau status in Supp. Table 1. All variables in regression models had a VIF < 5 and a tolerance > 0.2, which are not considered to signal problematic levels of multicollinearity [60]. 

**Table 1:** Demographics and biomarker’s data

No.(% of total)	CU	MCI	AD
	132 (75%)	27 (25%)	17 (10%)
Sex (female%)	65.9%	48.2%	47.1%
Age, mean (SD), y	72.1 (5.8)	72.9 (4.1)	68.7 (7.0)
Education, mean (SD), y	15.3 (3.8)	15.4 (3.0)	14.9 (3.3)
*APOE* ε*4* status	25.8%^b, c^	55.6%^a^	52.9%^a^
Handedness (right%)	88.6%	92.6%	88.2%
[^18^F]MK6240 temporal meta-ROI SUVR, mean (SD)	0.96 (0.12)^b, c^	1.49 (0.53)^a^	2.1 (0.85)^a^
[^18^F]AZD4694 SUVR, mean (SD)	1.44 (0.35)^b, c^	2.38 (0.47)^a^	2.27 (0.39)^a^
Laterality Index (LI-tau)	0.001 (0.03)^c^	−0.007 (0.11)	−0.042 (0.06)^a^
Absolute Laterality Index (LI-tau)	0.016 (0.02)^b, c^	0.073 (0.08)^a^	0.05 (0.05)^a^

Post-hoc Dunn tests were conducted to assess differences between groups except for sex and APOE ε4 status, where contingency chi-square tests were applied. FDR correction for multiple comparisons were performed in all cases. Significant differences between diagnostic groups: aCU; bMCI; cAD. Abbreviations: *AD* Alzheimer’s disease, *CU* cognitively unimpaired, *LI-tau* Laterality Index for tau asymmetry, *MCI* mild cognitive impairment, *SUVR* Standardized Uptake Value Ratio, *SD* Standard Deviation

### Voxel-wise analysis

**Fig. 1:**
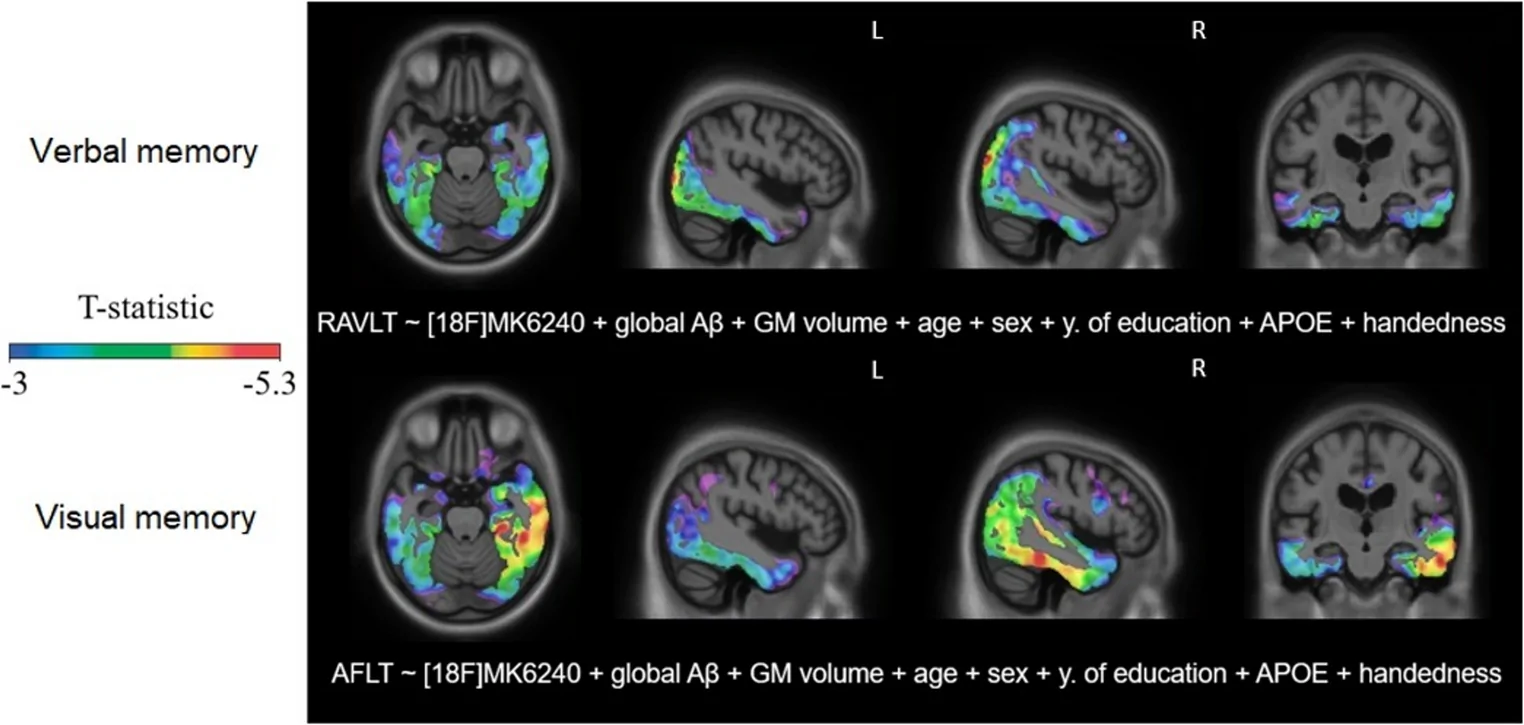
The association between tau load and visual memory scores predominates in the right temporal lobe (bottom). Verbal (top) memory scores are similarly related to tau loads bilaterally. T-statistical parametric maps were corrected for multiple comparisons using a random field theory cluster threshold of *p*<0.001, overlaid on the Alzheimer’s Disease Neuroimaging Initiative reference template. Two linear regression models were used, where either verbal delayed recall or visual delayed recall scores were entered as outcome variables and [^18^F]-MK6240 as predictor. Grey matter volume, age, sex, years of education, APOE status, handedness and amyloid-β positivity were used as covariates

Our voxel-wise analyses revealed that both types of memory had associations with tau in both hemispheres. The strength of the association was higher in the right hemisphere for visual memory performance (Fig. 1, random field theory corrected [61] at *p*<0.001). The strongest associations were found in lateral temporal and posterior cortical regions for both verbal and visual modalities.

### Simple regression models, LI-tval and LI-vox

**Fig. 2:**
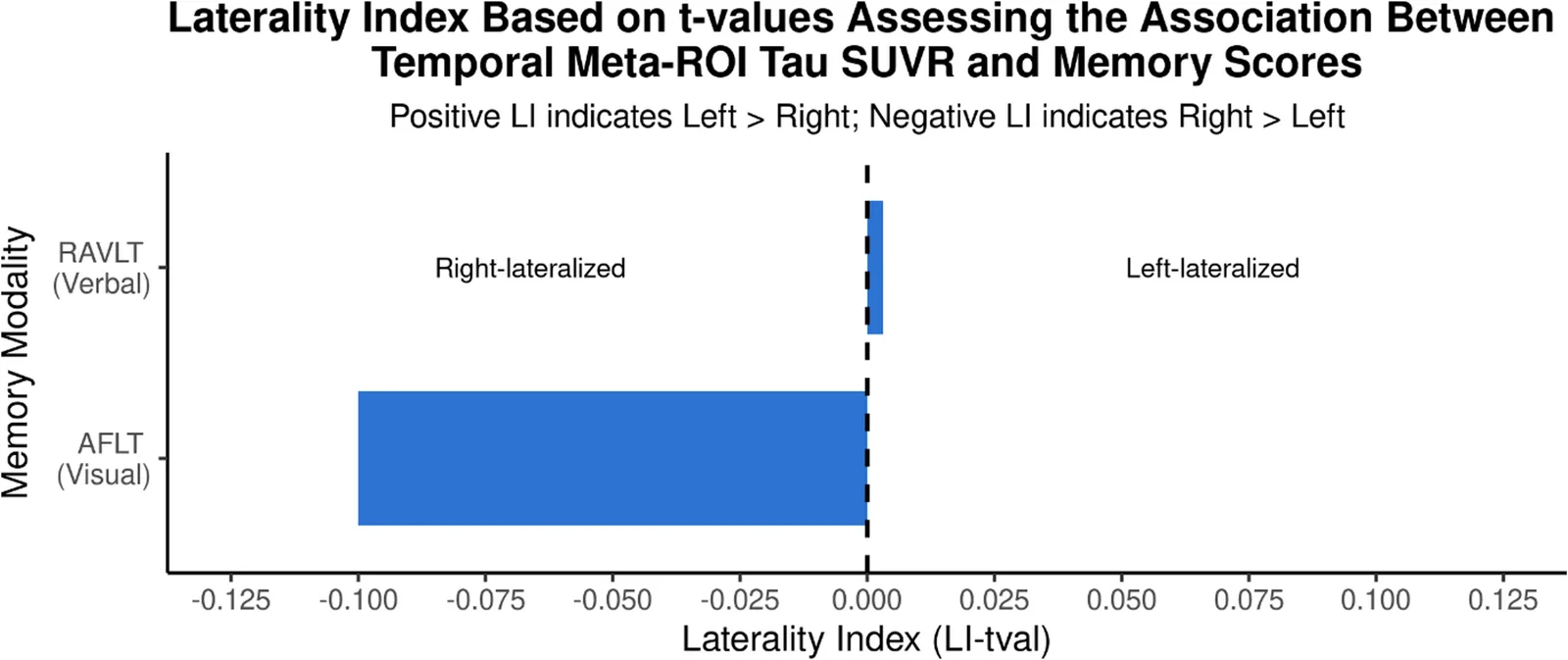
Visual memory is lateralized to the right. The LI-tval suggests the association between visual memory and tau SUVR that is lateralized. The LI-tval is calculated using the t-values of the associations between right or left temporal meta-ROI tau SUVR and verbal or visual memory scores. A negative value indicates a stronger association on the right hemisphere, and a positive value indicates a stronger association on the left hemisphere

**Fig. 3:**
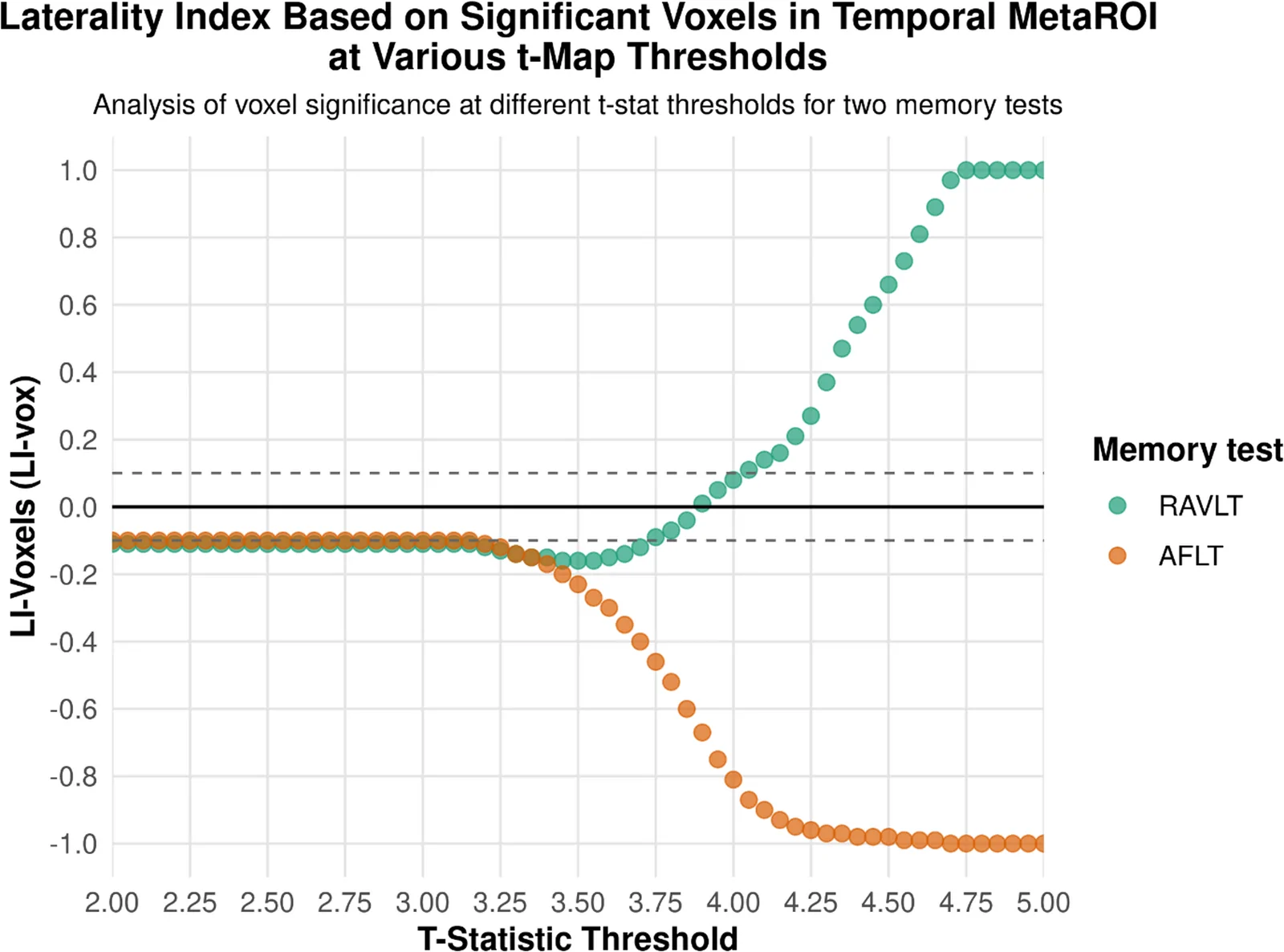
The strongest associations between tau and memory deficits are lateralized for both verbal memory (green) on the left and visual memory (blue) on the right. The LI-vox at increasing t-value thresholds shows that the associations with verbal memory are lateralized on the left, as indicated by positive values ≥ 0.1, while the associations with visual memory are lateralized on the right, as indicated by values ≤ −0.1. The plot indicates the number of voxels that remain significant across hemispheres at different t-values for the association between tau and verbal or visual memory scores

Subsequently, we investigated the independent association of temporal meta-ROI [^18^F]MK6240 SUVR with verbal and visual memory scores for each brain hemisphere. Simple regression models confirmed significant associations between tau SUVR and memory scores in all cases (*β*_*AFLT~Rtau*_=−0.39, *p* < 0.001; *β*_*AFLT~Ltau*_=−0.34, *p* < 0.001; *β*_*RAVLT~Rtau*_=−0.29, *p* < 0.001; *β*_*RAVLT~Ltau*_=−0.30, *p* < 0.001). We controlled for the effect of amyloid-β SUVR, grey matter volume, age, APOE status, sex, years of education, and handedness. Full model statistics are available in Supp. Tables 2 and 3. Moreover, the LI-tval was near 0 for verbal memory (LI-tval_RAVLT_: 0.003). Meanwhile, the LI-tval for visual memory (LI-tval_AFLT_: −0.1) indicated stronger associations between memory and tau in the right hemisphere; as previously mentioned, values that reach ± 0.1 are considered to signal significant lateralization [57] (Fig. 2). Furthermore, the LI-vox_RALT_ showed lateralization on the right at lower thresholds, but a shift to lateralization on the left beginning at around *t* = 3.75 (Fig. 3). The LI-vox_AFLT_ confirmed right lateralization across t-value thresholds, with a sharp decrease in LIs (i.e., further right lateralization) starting at around *t* = 3.5 (Fig. 3).

**Table 2:** Regression models’ parameters for the association with verbal memory scores when controlling for visual memory scores for temporal meta-ROI SUVR

Rey Auditory Verbal Learning Test (verbal memory z-scores)			
Coefficients	Beta (95% CI)	T-value	*P* value
R tau temporal meta-ROI SUVR	−0.1 (−0.23-0.03)	−1.512	0.132
[^18^F]AZD4694 SUVR, mean (SD)	−0.22 (−0.33- −0.1)	−3.544	**< 0.001**
Grey matter volume (residuals)	0.13 (0.03–0.23)	2.549	**0.012**
AFLT DR z-score	0.5 (0.38–0.62)	8.134	**< 0.001**
Years of education	0.09 (−0.01-0.19)	1.844	0.067
Age	−0.08 (−0.18-0.03)	−1.481	0.14
Handedness (left)	0.08 (−0.23-0.39)	0.527	0.599
Sex (female)	0.36 (0.16–0.55)	3.544	**< 0.001**
APOE4 status (positive)	−0.08 (−0.29-0.13)	−0.741	0.46
Rey Auditory Verbal Learning Test (verbal memory z-scores)			
Coefficients	Beta (95% CI)	T-value	*P* value
L tau temporal meta-ROI SUVR	−0.14 (−0.27- −0.01)	−2.105	**0.037**
[^18^F]AZD4694 SUVR, mean (SD)	−0.19 (−0.31- −0.07)	−3.072	**< 0.001**
Grey matter volume (residuals)	0.13 (0.03–0.23)	2.508	**0.013**
AFLT DR z-scores	0.49 (0.38–0.61)	8.298	**< 0.001**
Years of education	0.09 (−0.01-0.19)	1.935	0.055
Age	−0.09 (−0.19-0.02)	−1.674	0.096
Handedness (left)	0.07 (−0.24-0.37)	0.436	0.663
Sex (female)	0.37 (0.17–0.56)	3.665	**< 0.001**
APOE4 status (positive)	−0.07 (−0.28-0.14)	−0.63	0.529

Standardized β coefficients for predictor and covariates. Abbreviations: *R* right, *L* left, *SUVR* Standardized Uptake Value Ratio, *SD* Standard Deviation

**Table 3:** Regression models’ parameters for the association with visual memory scores when controlling for verbal memory scores for temporal meta-ROI SUVR

Aggie Figures Learning Test (visual/nonverbal memory z-scores)			
Coefficients	Beta (95% CI)	T-value	*P* value
R tau temporal meta-ROI SUVR	−0.22 (−0.36- −0.09)	−3.259	**0.001**
[^18^F]AZD4694 SUVR, mean (SD)	−0.06 (−0.19-0.07)	−0.891	0.374
Grey matter volume	0.01 (−0.11-0.11)	0.005	0.996
RAVLT DR z-scores	0.57 (0.43–0.71)	8.134	**< 0.001**
Years of education	0.05 (−0.06-0.15)	0.927	0.355
Age	−0.07 (−0.18-0.04)	−1.178	0.24
Handedness (left)	−0.18 (−0.51-0.15)	−1.062	0.29
Sex (female)	−0.2 (−0.42-0.01)	−1.848	0.066
APOE4 status (positive)	0.03 (−0.2-0.26)	0.265	0.791
Aggie Figures Learning Test (visual/nonverbal memory z-scores)			
Coefficients	Beta (95% CI)	T-value	*P* value
L tau temporal meta-ROI SUVR	−0.16 (−0.3- −0.01)	−2.182	**0.031**
[^18^F]AZD4694 SUVR, mean (SD)	−0.08 (−0.21-0.06)	−1.08	0.282
Grey matter volume	0.01 (0.1–0.12)	0.224	0.823
RAVLT DR z-scores	0.6 (0.45–0.74)	8.298	**< 0.001**
Years of education	0.05 (−0.06-0.16)	0.95	0.344
Age	−0.05 (−0.17-0.06)	−0.916	0.361
Handedness (left)	−0.19 (−0.52-0.15)	−1.089	0.278
Sex (female)	−0.22 (−0.44-0.01)	−1.901	0.059
APOE4 status (positive)	0.02 (−0.21-0.26)	0.206	0.837

Standardized β coefficients for predictor and covariates. Abbreviations: *R* right, *L* left, *SUVR* Standardized Uptake Value Ratio, *SD* Standard Deviation

### Combined regression models and across Braak stages

**Fig. 4:**
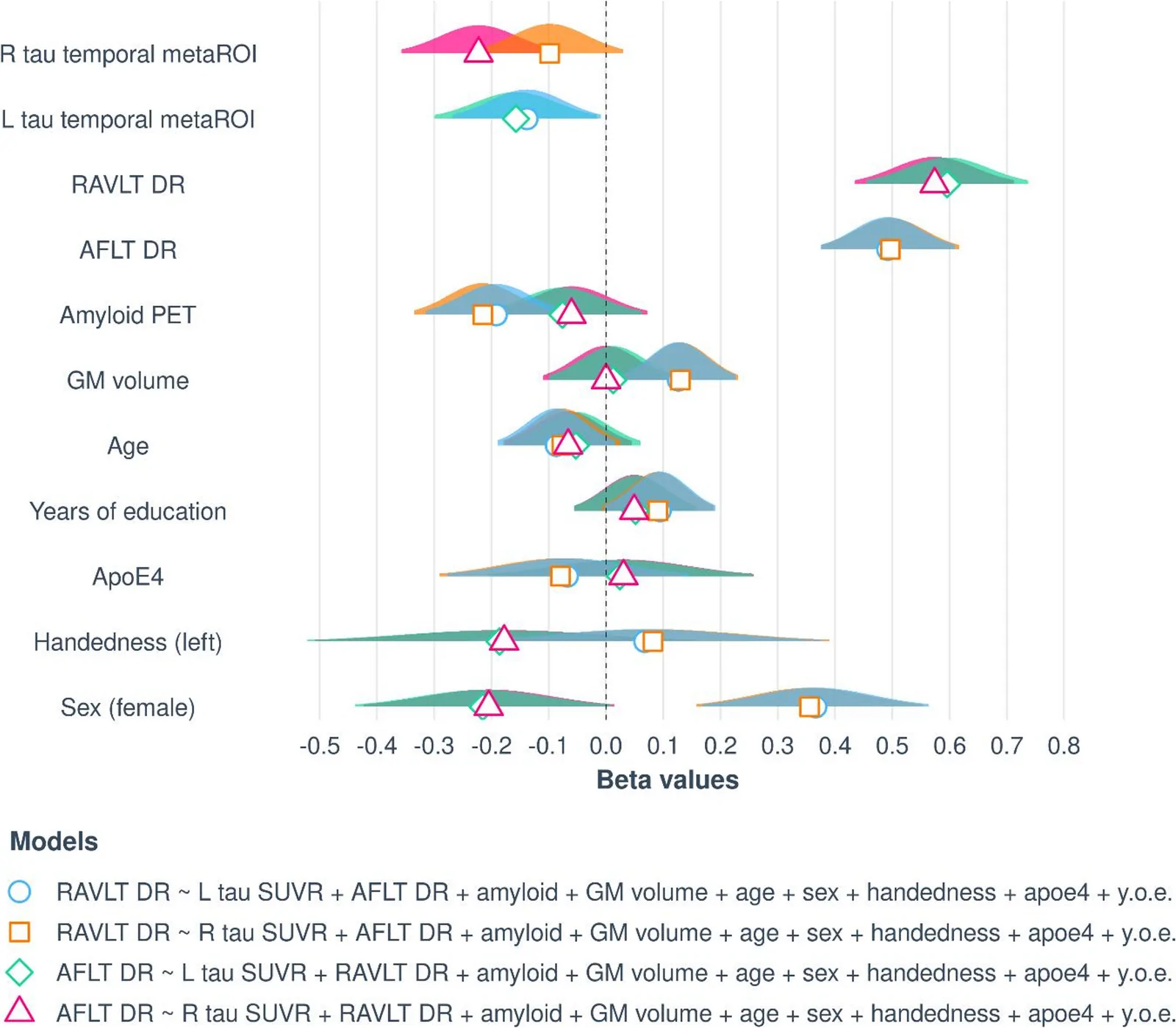
Tau load on the right temporal lobe is primarily linked to visual memory deficits. The plot displays standardized *ß* values (x-axis) for parameters (y-axis) in combined regression models with either verbal (RAVLT) or visual (AFLT) memory scores as outcome variable. Models are color and shape coded. Blue circle: association between verbal memory and tau SUVR on the left. Orange square: association between verbal memory and tau SUVR on the right. Turquoise diamond: association between visual memory and tau SUVR on the left. Pink triangle: association between visual memory and tau SUVR on the right. The only surviving association between tau temporal meta-ROI [^18^F]MK6240 SUVR and memory in the right hemisphere is that for visual memory

In regression models including verbal or visual memory scores as covariates, associations with right temporal meta-ROI [^18^F]MK6240 SUVR were significant only for visual memory (*β*_*AFLT~Rtau*_=−0.22, *p* = 0.001; *β*_*RAVLT~Rtau*_=−0.1, *p* = 0.13); while associations with left temporal meta-ROI [^18^F]MK6240 SUVR remained significant for both tests (*β*_*AFLT~Ltau*_=−0.16, *p* = 0.03; *β*_*RAVLT~Ltau*_=−0.14, *p* = 0.04). The results of these models are represented in Fig. 4, and their statistical results can be found in Tables 2 and 3. Combined models were also applied using right or left SUVR from PET-Braak stages. Visual memory was significantly associated with tau SUVR in the right hemisphere in all but Braak II and Braak V stages, and in the left hemisphere in Braak III and IV. Verbal memory was significantly associated with tau SUVR in left Braak I and left Braak III. Full model results for regression analyses across PET-Braak stages can be found in Supp. Tables 4–15.

### Tau asymmetry

**Fig. 5:**
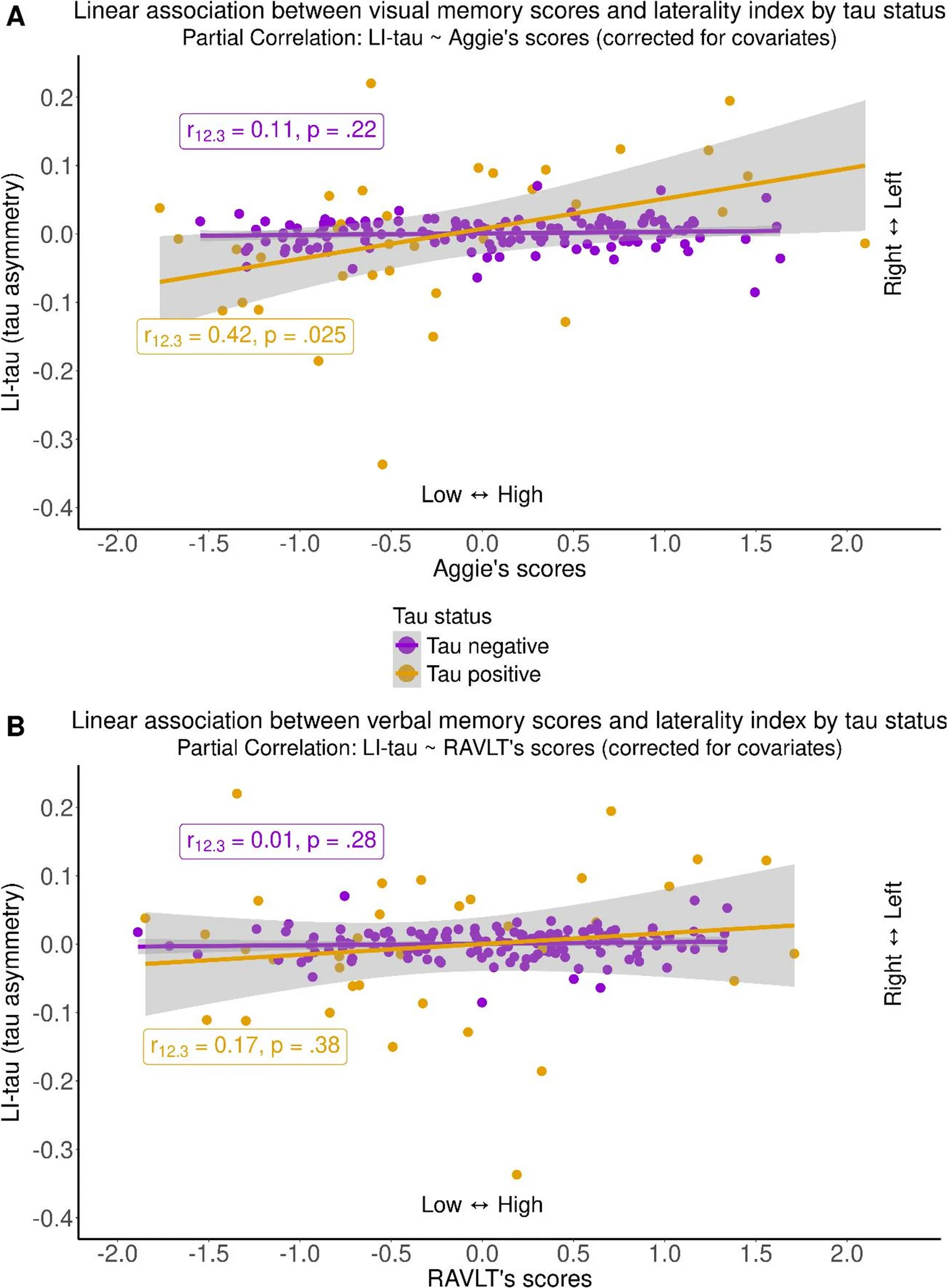
The magnitude of right tau asymmetry is associated with worse visual memory scores in tau positive individuals. Association between laterality index and (**A**) visual delayed recall scores and (**B**) verbal delayed recall scores by tau status. Negative index scores indicate right asymmetry, while positive index scores indicate left asymmetry. Scatterplots display the relationship between residuals of the laterality index and residuals of memory scores when correcting for amyloid-β SUVR, grey matter volume, age, sex, years of education, APOE status and handedness

We also tested the hypothesis that the LI-tau, which reflects tau asymmetry between brain hemispheres, would be related to both visual and verbal memory performance. The LI-tau was significantly associated with visual memory scores (*r*_*12.3*_=0.21, *p* = 0.005), but there was no significant correlation between the LI-tau and verbal memory scores (*r*_*12.3*_=0.09, *p* = 0.25) (Supp. Figure 2). Further analyses were conducted to assess what was driving the significant association involving visual recall scores, and to examine potential associations with verbal recall scores in sub-groups. When segregating participants by cognitive status, our results showed that the relationship between tau and visual memory performance was no longer significant in either cognitively unimpaired elderly or CI individuals (CU: *r*_*12.3*_=0.05, *p* = 0.58; CI: *r*_*12.3*_=0.3, *p* = 0.07) (Supp. Figure 3). However, the effect was marginally significant within the CI group. Consistent with the result for the whole sample, no significant association was found for verbal memory (CU: *r*_*12.3*_=0.06, *p* = 0.51; CI: *r*_*12.3*_=−0.03, *p* = 0.87). Participants who were classified as tau-positive exhibited a significant relationship between the LI-tau and visual memory scores (*r*_*12.3*_=0.42, *p* = 0.025), in contrast to tau-negative individuals (*r*_*12.3*_=0.11, *p* = 0.22). The relationship between the LI-tau and verbal memory scores remained nonsignificant for both groups (Tau-: *r*_*12.3*_=0.1, *p* = 0.28; Tau+: *r*_*12.3*_=0.17, *p* = 0.38) (Fig. 5). Moreover, our findings unveiled a significant correlation between the LI-tau and visual memory scores in subjects that had significant tau in any of the PET-Braak stages (*r*_*12.3*_=0.27, *p* = 0.024), but this relationship was absent in individuals with no significant tau in any of the PET-defined Braak regions (*r*_*12.3*_=0.09, *p* = 0.37). Consistent with previous results, no significant association was detected for verbal memory deficits (Braak 0: *r*_*12.3*_=0.08, *p* = 0.44; Braak I-VI: *r*_*12.3*_=0.11, *p* = 0.36) (Supp. Figure 4). Finally, we conducted the analysis classifying participants into APOE4 carriers and non-carriers, and we found that the associations between the LI-tau and visual memory were significant or marginal in APOE4 non-carriers and carriers, respectively (APOE4-: *r*_*12.3*_=0.19, *p* = 0.04; APOE4+: *r*_*12.3*_=0.24, *p* = 0.06) (Supp. Figure 5). Once again, the associations with verbal memory were nonexistent (APOE4-: *r*_*12.3*_=0.07, *p* = 0.44; APOE4+: *r*_*12.3*_=0.11, *p* = 0.4). All partial correlation analyses were corrected for the covariates described in regression analyses.

## Discussion

Our investigation aimed to evaluate whether the relationship between tangles and memory deficits was lateralized with respect to verbal and visual modalities in Alzheimer’s disease. Our findings revealed a degree of lateralization in the visual modality, while verbal memory exhibited a reduced degree of lateralization. Voxel-wise analyses demonstrated lateralization in the right hemisphere for the visual modality. Interestingly, the larger the magnitude of the association between tau and verbal memory, the larger its lateralization to the left hemisphere. Regression models including both verbal and visual memory scores confirmed that the relationship between visual memory and tau load in the right hemisphere was stronger than that between verbal memory and tau. Moreover, hemispheric asymmetry of tau load was significantly associated with visual delayed recall performance, but not with verbal delayed recall performance. Our results partially support well-established literature pointing at hemispheric lateralization of the recall function reported in other brain pathologies [8]. 

Voxel-wise analyses revealed that the association between [^18^F]MK6240 SUVR and recall performance was stronger in the right cerebral hemisphere for visual recall (Fig. 1), and not apparent for verbal recall. When examining tau SUVR from the temporal meta-ROI by hemisphere, we found that both standardized *β* coefficients and t-values were higher for the relationship between visual memory and tau in the right hemisphere, but similar across hemispheres for verbal memory (Supp. Table 3; Fig. 2 for the LI-tval). However, lateralization of the association was clearly revealed at higher t-values in both cases (Fig. 3). This can be explained as voxel-based approaches are exempted from a priori anatomical boundaries. Furthermore, when both verbal and visual memory scores were entered in regression models using tau temporal meta-ROI SUVR, it was only the relationship between visual memory and tau that survived in the right hemisphere, while scores from both tests were significantly associated with tau in the left hemisphere (Fig. 4). A similar pattern was found across Braak regions, with the notable exception of Braak I, where we found that only the association between tau and verbal memory scores in the left and visual memory scores in the right remained significant. Our findings thus support widespread lateralization of the association between tangles and visual recall in the right hemisphere, while voxels with strong associations between tangles and verbal recall lateralize to the left.

The apparent lack of lateralization involving verbal memory deficits stands in opposition to a wealth of evidence from lesion [62] and MRI studies [11, 63]. In addition, people with high tau load showed a tendency towards increased tau in the right hemisphere in our sample (Supp. Figure 1). Previous research has demonstrated that the absolute LI correlates with tau accumulation [56], a finding we replicated (Supp. Figure 2). Hence, results for lateralization of verbal recall might be unexpected within the framework described above. Nonetheless, a more detailed scrutiny of these associations is warranted. Voxel-wise contrasts (LI-vox; Fig. 3) revealed some lateralization in the unexpected direction (the right) when t-values were low, but overwhelming lateralization in the expected direction (the left) when t-values were high. Therefore, the bilaterality of the association in broader areas may reflect a compensatory effect of lateralization to the right at low t-values and lateralization to the left at high t-values. More research is needed to determine whether these findings are specific to our sample or could be generalized.

Despite revealing lateralized associations, voxel-wise analyses demonstrated that these involved similar brain areas regardless of modality. It should be noted that this relationship was not limited to the medial temporal lobe, although this is the dominant feature possibly due to the preponderance of tau pathology in temporal regions in Alzheimer’s disease [64]. Higher tau in large swaths of medial, lateral and inferior temporal, as well as posterior neocortical and some frontal regions was associated with worse performance beyond the effect of amyloid and grey matter volume. These are regions known to be vulnerable to Alzheimer’s disease pathology [31, 65]. The link between tau load in the temporal lobe and memory deficits has been well established [66]. The additional regions implicated in our study include posterior regions that typically display Alzheimer’s disease-related tauopathy [31] and that are known to be relevant to recall functions [67]. Multiple studies have shown that tau load in all these regions is associated to lower performance in recall tests [3, 34, 41]. 

Finally, our results indicate that tau’s hemispheric asymmetry was significantly related to visual memory scores, but not to verbal memory performance. This finding was particularly pronounced in individuals who were classified as tau-positive (Tau+_metaROI_: *r*_*12.3*_=0.42, *p* = 0.025; Tau + _BI_: *r*_*12.3*_=0.27, *p* = 0.024; whole sample: *r*_*12.3*_=0.21, *p*=0.005), indicating that this relationship was driven by subjects with tau spread beyond the MTL. Most studies in the context of Alzheimer’s disease have sought to explore hemispheric asymmetry across atypical forms of AD, where left asymmetry is often observed in patients with primary progressive aphasia [41]. A recent study demonstrated that extreme right asymmetry was linked to behavioral problems, while extreme left asymmetry was related to worse language performance [56]. Our study is the first to reveal that tau asymmetry is associated with visual memory performance.

Lateralization of language and visuospatial functions constitutes a feature of the human brain [30]. Much of the research regarding lateralization of deficits in recall function has been carried out with epileptic patients that had unilateral anterior MTL resection [68], patients with brain injury [62], and with participants with different cognitive profiles in MRI and PET studies [8, 21]. Studies including patients with resection or lesions of anterior portions of the temporal lobe have frequently found that lesions on the right side are related to difficulties with visuospatial material, while lesions on the left are associated with difficulties with verbal material [62, 68]. In healthy individuals, neuroimaging studies have shown that activation in right frontal or temporal cortices is linked to encoding and/or retrieval of visuospatial material, whereas activation on the left side is associated with encoding and/or retrieval of verbal material [8, 11, 21, 63]. 

Yet, clinical studies addressing functional lateralization remain controversial [69]. Some weaknesses include using models of brains that may have reorganized due to pathology or insult [70], a lack of standardized procedures to measure visual or nonverbal recall [12], or the challenge of developing tests that validly measure nonverbal memory outcomes [71]. In our study, some of these challenges may have been attenuated by using the AFLT and the RAVLT, which allow for a direct comparison between visual and verbal modalities. Research where both tests have been compared has confirmed a double dissociation in patients with temporal resection [39], albeit these differences were not confined to delayed recall. Our recent study using hippocampal subfields has also reported that AFLT scores correlated with lower volume in certain sub-regions of the right hippocampus in cognitively unimpaired and mildly cognitively impaired elderly [72]. Furthermore, the AFLT contains figures that are not easily verbalized [21]. 

Studies addressing lateralization of cognitive deficits in Alzheimer’s disease have been more limited. Some structural MRI studies have hinted at material-specific lateralization, with associations between left and right volumes of MTL structures and verbal or visual memory scores, respectively [73, 74]. Regarding the association between amyloid PET and memory deficits, some researchers have found an association between amyloid levels in the left hemisphere and verbal memory performance, but not between visual memory and amyloid load in the right hemisphere [75]. Others have found that amyloid aggregates mediated the left-lateralized association between posterior Default Mode Network connectivity and verbal memory scores [76]. However, these studies preceded the development of tau PET imaging. More recent literature has found no lateralization of the association between cognition and amyloid load [77]. With respect to tau, a few tau studies have shown lateralization of deficits as a function of tangle load [34, 35], but others have not found such associations [2, 3, 32, 33]. 

Our findings have implications for clinicians and also for research. Healthcare professionals may need to reconsider indiscriminately administering memory tests of different sorts to measure the degree of memory impairment at a certain stage of the disease. For example, recognition tests may not be as efficient as delayed recall tests when early stage pathology is suspected [78]. Similarly, tests that are based on images that are easily verbalized may not accurately represent visual/nonverbal deficits. As mentioned in the introduction, research may be positively impacted by a more detailed approach to cognitive deficits. For instance, clinical trials that use mild memory deficits as an outcome measure related to early accumulation of tau may obtain more informative results if they focus on measures of delayed recall. In the case of the present findings, we have seen that men tend to perform worse in verbal delayed recall when compared to women, but there appear to be no significant differences between sexes for visual memory.

The present conclusions are limited by several methodological considerations. First, the TRIAD cohort is a convenience sample consisting of participants who were motivated to enroll in Alzheimer’s disease studies and is therefore subject to recruitment and sampling biases.

Second, the evidence we present in support of lateralization is not unequivocal. There is no straightforward statistical method to compare the strength of associations between memory modalities and tau across hemispheres; however, including both scores in the same regression models provides a rigorous framework for testing these hypotheses. We also acknowledge that, despite having included them in our analyses, other variables may have influenced our results.

One of these variables is handedness. To address this concern, we repeated the analyses in right-handed individuals only, and the results were nearly identical to (and, if anything, more clearly visualized than) those obtained in the full sample. This additional analysis is now included in the supplementary materials (Supp. Figure 8).

Another potential confounder is amyloid, which displayed strong associations with memory in our study. These likely reflect sample composition: cognitively impaired individuals who are exclusively amyloid-positive, most of whom are not in the late stages of the disease. In later AD stages, amyloid deposition reaches a plateau [79], reducing variability and potentially truncating correlations with memory scores, whereas our sample includes a large proportion of individuals with low (CU Aβ-) and moderate (CU Aβ + and MCI) levels of amyloid. While some studies report associations between amyloid burden and cognitive performance [80], these findings should be interpreted with caution. For example, studies relying on amyloid PET without concurrent tau PET measures may, at least in part, reflect effects of unmodeled tau. Consistent with this interpretation, a substantial body of work indicates that tau pathology shows stronger and more spatially specific associations with cognitive performance [81], particularly memory [82], while amyloid may play a more permissive or upstream role [83, 84]. Notably, the combination of amyloid and tau pathology is associated with greater cognitive impairment than either pathology alone [85]. 

Another contributing factor to the relative strength of the associations between amyloid PET and cognitive performance might be that our tau measurement derives from the temporal meta-ROI, which reflects more advanced pathology. Literature suggests that associations between tau and memory are strongest when using regions of early tau deposition [86]. These considerations are supported by our supplementary analyses, where the associations between amyloid PET and memory scores are reduced in a larger sample including CI individuals that are amyloid-β negative (Supp. Figure 4) and when using Braak Stage I SUVR in the same sample as a measurement of tau (Supp. Figure 5).

Taken together, these findings indicate that neither handedness nor amyloid-β SUVR substantially influence the observed lateralization patterns, and that apparent differences in associations across hemispheres or memory modalities are better interpreted in the context of sample characteristics, regional tau measurement, and the interdependence of verbal and visual memory scores.

Moreover, the finding regarding Braak I should be interpreted with caution. An association that is stronger between left tau and verbal memory than between left tau and visual memory and vice-versa may not be indicative of the former being also stronger than the association between right tau and verbal memory. Indeed, we discovered that the strength of the association between tau and verbal memory is comparable across hemispheres (Supp. Figure 11). The results may indicate the unequal contribution of other variables in the combined model.

In addition, the transentorhinal cortex is a very small region, but PET offers limited spatial resolution for small structures. Furthermore, although we outlined the advantages of using the AFLT as an analog of the RAVLT, a recent meta-analysis reported that face memory may be more sensitive to right hippocampal damage than design memory in epilepsy populations [18]. If this finding extends to Alzheimer’s disease neuropathology, more conclusive results might have emerged. Lastly, identifying linear relationships between indicators of tau asymmetry and test performance may itself be difficult, although our data partially aligned with our initial expectations.

Our study provides a unique opportunity to contribute to the debate about the clinical heterogeneity of Alzheimer’s disease based on anatomical-clinical evidence. Overall, we found that the link between tangles and verbal recall deficits is less clearly lateralized than that observed for visual recall, which exhibits more pronounced hemispheric specificity. The findings may have clinical implications, as deficits on memory tests of different modalities reflect distinct underlying neuropathology and therefore should not be considered interchangeably. To conclude, our findings provide evidence supporting the lateralization of memory deficits as a function of tau tangle load.

## Supplementary Information

Below is the link to the electronic supplementary material.


Supplementary Material 1 (DOCX 3.35 MB)


## Data Availability

The data presented in this study are available from the corresponding author upon reasonable request, and such arrangements are subject to standard data-sharing agreements.
