# Supplementary material for: Hemispheric lateralization of Tau associations with visual and verbal memory in Alzheimer’s disease
Source: Eur J Nucl Med Mol Imaging. 2026 Jun 5;53(11):6195–211. doi: 10.1007/s00259-026-07898-z (PMC13525940; doi:10.1007/s00259-026-07898-z)
Supplement: Supplementary file 1 [file 259_2026_7898_MOESM1_ESM.docx]

**Supplementary Figure 1.** Date differences in days across scans and neuropsychological tests

**Supplementary Table 1.** Demographics and biomarker’s data stratified by tau status

|  | Tau - | Tau + | P-value |
| --- | --- | --- | --- |
| No.(% of total) | 141 (80%) | 35 (20%) |  |
| Sex (female%) | 61.7% | 60% | 0.853 |
| Age, mean (SD), y | 72.1 (5.77) | 71.1 (5.77) | 0.643 |
| Education, mean (SD), y | 15.3 (3.7) | 15.1 (3.2) | 0.984 |
| *APOE* ε*4* status | 26.2% | 60% | **<0.001** |
| Handedness (right%) | 89% | 91% | 0.636 |
| [^18^F]MK6240 temporal meta-ROI SUVR, mean (SD) | 0.96 (0.1) | 1.95 (0.67) | **<0.001** |
| [^18^F]AZD4694 SUVR, mean (SD) | 1.49 (0.39) | 2.38 (0.42) | **<0.001** |
| Laterality Index (LI-tau) | -0.004 (0.02) | -0.009 (0.11) | 0.916 |
| Absolute Laterality Index (LI-tau) | 0.015 (0.16) | 0.083 (0.07) | **<0.001** |

Post-hoc Dunn tests were conducted to assess differences between groups except for sex and APOE ε4 status, where contingency chi-square tests were applied. FDR correction for multiple comparisons were performed in all cases. Abbreviations: LI-tau, Laterality Index for tau asymmetry; SUVR: Standardized Uptake Value Ratio; SD: Standard Deviation.

**Supplementary Table 2.** Regression models’ parameters for the independent association between verbal memory scores and tau SUVR

| Rey Auditory Verbal Learning Test (verbal memory z-scores) | | | |
| --- | --- | --- | --- |
| Coefficients | Beta (95% CI) | T-value | *P* value |
| R tau temporal meta-ROI SUVR | -0.29 (-0.43- -0.15) | -4.093 | **<0.001** |
| [^18^F]AZD4694 SUVR, mean (SD) | -0.34 (-0.48- -0.21) | -4.956 | **<0.001** |
| Grey matter volume | 0.18 (0.06-0.3) | 3.051 | **0.003** |
| Years of education | 0.16 (0.05-0.27) | 2.823 | **0.005** |
| Age | -0.15 (-0.27- -0.03) | -2.547 | **0.012** |
| Handedness (left) | -0.01 (-0.12-0.11) | -0.046 | 0.963 |
| Sex (female) | 0.36 (0.12-0.59) | 3.002 | **0.003** |
| APOE4 status (positive) | -0.04 (-0.16-0.08) | -0.711 | 0.478 |
| Rey Auditory Verbal Learning Test (verbal memory z-scores) | | | |
| Coefficients | Beta (95% CI) | T-value | *P* value |
| L tau temporal meta-ROI SUVR | -0.3 (-0.45- -0.16) | -4.118 | **<0.001** |
| [^18^F]AZD4694 SUVR, mean (SD) | -0.32 (-0.46- -0.18) | -4.531 | **<0.001** |
| Grey matter volume | 0.19 (0.07-0.3) | 3.174 | **0.002** |
| Years of education | 0.17 (0.06-0.28) | 2.979 | **0.003** |
| Age | -0.16 (-0.28- -0.04) | -2.630 | **0.009** |
| Handedness (left) | -0.01 (-0.12-0.1) | -0.182 | 0.859 |
| Sex (female) | 0.37 (0.13- 0.6) | 3.109 | **0.002** |
| APOE4 status (positive) | -0.04 (-0.15-0.08) | -0.618 | 0.537 |

Standardized β coefficients for predictor and covariates. Top: parameters for the model including right tau SUVR as predictor. Bottom: parameters of the model including left tau SUVR as predictor. Abbreviations: R, right; L, left; SUVR, Standardized Uptake Value Ratio; CI, confidence interval; SD: Standard Deviation.

**Supplementary Table 3.** Regression models’ parameters for the independent association between visual/nonverbal memory scores and tau SUVR

| Aggie Figures Learning Test (visual/nonverbal memory z-scores) | | | |
| --- | --- | --- | --- |
| Coefficients | Beta (95% CI) | T-value | *P* value |
| R tau temporal meta-ROI SUVR | -0.39 (-0.54- -0.24) | -5.085 | **<0.001** |
| [^18^F]AZD4694 SUVR, mean (SD) | -0.26 (-0.4- -0.11) | -3.455 | **<0.001** |
| Grey matter volume (residuals) | 0.11 (-0.02-0.23) | 1.633 | 0.104 |
| Years of education | 0.14 (0.02-0.26) | 2.312 | **0.022** |
| Age | -0.15 (-0.28- -0.03) | -2.378 | **0.019** |
| Handedness (left) | -0.06 (-0.18-0.06) | -0.925 | 0.356 |
| Sex (female) | -0.01 (-0.25-0.25) | -0.006 | 0.995 |
| APOE4 status (positive) | -0.01 (-0.14-0.12) | -0.154 | 0.878 |
| Aggie Figures Learning Test (visual/nonverbal memory z-scores) | | | |
| Coefficients | Beta (95% CI) | T-value | *P* value |
| L tau temporal meta-ROI SUVR | -0.34 (-0.5- 0.18) | -4.161 | **<0.001** |
| [^18^F]AZD4694 SUVR, mean (SD) | -0.27 (-0.42- -0.11) | -3.419 | **<0.001** |
| Grey matter volume (residuals) | 0.13 (0.01-0.25) | 1.913 | 0.058 |
| Years of education | 0.15 (0.03-0.28) | 2.435 | **0.016** |
| Age | -0.15 (-0.28- -0.02) | -2.213 | **0.028** |
| Handedness (left) | -0.06 (-0.19-0.06) | -1.017 | 0.311 |
| Sex (female) | 0.01 (-0.25-0.26) | 0.034 | 0.973 |
| APOE4 status (positive) | -0.011 (-0.14-0.12) | -0.161 | 0.873 |

Standardized β coefficients for predictor and covariates. Top: parameters for the model including right tau SUVR as predictor. Bottom: parameters of the model including left tau SUVR as predictor. Abbreviations: R, right; L, left; SUVR, Standardized Uptake Value Ratio; CI, confidence interval; SD: Standard Deviation.

**Supplementary Table 4.** Regression models’ parameters for the association with verbal memory scores when controlling for visual memory scores for Braak I SUVR.

| Rey Auditory Verbal Learning Test (verbal memory z-scores) | | | |
| --- | --- | --- | --- |
| Coefficients | Beta (95% CI) | T-value | *P* value |
| R Braak I SUVR | -0.13 (-0.27-0.01) | -1.895 | 0.06 |
| [^18^F]AZD4694 SUVR, mean (SD) | -0.18 (-0.31- -0.04) | -2.616 | **0.01** |
| Grey matter volume (residuals) | 0.15 (0.05-0.24) | 2.911 | **0.004** |
| AFLT DR z-score | 0.5 (0.38-0.61) | 8.44 | **<0.001** |
| Years of education | 0.09 (-0.01-0.19) | 1.912 | 0.06 |
| Age | -0.07 (-0.16-0.03) | -1.305 | 0.194 |
| Handedness (left) | 0.08 (-0.23-0.39) | 0.511 | 0.61 |
| Sex (female) | 0.36 (0.16-0.56) | 3.6 | **<0.001** |
| APOE4 status (positive) | -0.05 (-0.27-0.16) | -0.478 | 0.633 |
| Rey Auditory Verbal Learning Test (verbal memory z-scores) | | | |
| Coefficients | Beta (95% CI) | T-value | *P* value |
| L Braak I SUVR | -0.15 (-0.29- -0.01) | -2.084 | **0.039** |
| [^18^F]AZD4694 SUVR, mean (SD) | -0.17 (-0.3- -0.03) | -2.485 | **0.014** |
| Grey matter volume (residuals) | 0.14 (0.05-0.24) | 2.909 | **0.004** |
| AFLT DR z-scores | 0.5 (0.38-0.61) | 8.427 | **<0.001** |
| Years of education | 0.1 (0.01-0.19) | 1.974 | **0.05** |
| Age | -0.07 (-0.16-0.03) | -1.335 | 0.184 |
| Handedness (left) | 0.07 (-0.24-0.37) | 0.428 | 0.669 |
| Sex (female) | 0.37 (0.17-0.57) | 3.671 | **<0.001** |
| APOE4 status (positive) | -0.04 (-0.26-0.17) | -0.383 | 0.702 |

Standardized β coefficients for predictor and covariates. Abbreviations: AFLT DR, Aggie Figures Learning Test Delayed Recall; L, left; R, right; SD, Standard Deviation; SUVR, Standardized Uptake Value Ratio.

**Supplementary Table 5.** Regression models’ parameters for the association with visual memory scores when controlling for verbal memory scores for Braak I SUVR

| Aggie Figures Learning Test (visual/nonverbal memory z-scores) | | | |
| --- | --- | --- | --- |
| Coefficients | Beta (95% CI) | T-value | *P* value |
| R Braak I SUVR | -0.16 (-0.31- -0.01) | -2.044 | **0.043** |
| [^18^F]AZD4694 SUVR, mean (SD) | -0.06 (-0.21-0.09) | -0.736 | 0.463 |
| Grey matter volume | 0.03 (-0.08-0.14) | 0.598 | 0.551 |
| RAVLT DR z-scores | 0.6 (0.46-0.74) | 8.44 | **<0.001** |
| Years of education | 0.05 (-0.06-0.16) | 0.93 | 0.354 |
| Age | -0.03 (-0.14-0.08) | -0.513 | 0.61 |
| Handedness (left) | -0.17 (-0.51-0.16) | -1.021 | 0.309 |
| Sex (female) | -0.22 (-0.45- -0.01) | -1.986 | **0.049** |
| APOE4 status (positive) | 0.04 (-0.19-0.28) | 0.369 | 0.713 |
| Aggie Figures Learning Test (visual/nonverbal memory z-scores) | | | |
| Coefficients | Beta (95% CI) | T-value | *P* value |
| L Braak I SUVR | -0.15 (-0.3-0.01) | -1.889 | 0.061 |
| [^18^F]AZD4694 SUVR, mean (SD) | -0.06 (-0.21-0.09) | -0.801 | 0.424 |
| Grey matter volume | 0.03 (-0.08-0.14) | 0.587 | 0.558 |
| RAVLT DR z-scores | 0.6 (0.46-0.75) | 8.427 | **<0.001** |
| Years of education | 0.05 (-0.06-0.16) | 0.964 | 0.337 |
| Age | -0.03 (-0.14-0.08) | -0.504 | 0.615 |
| Handedness (left) | -0.18 (-0.52-0.15) | -1.08 | 0.282 |
| Sex (female) | -0.22 (-0.44-0.01) | -1.942 | 0.054 |
| APOE4 status (positive) | 0.05 (-0.19-0.28) | 0.382 | 0.703 |

Standardized β coefficients for predictor and covariates. Abbreviations: L, left; R, right; RAVLT DR, Rey Auditory Verbal Learning Test Delayed Recall; SD, Standard Deviation; SUVR, Standardized Uptake Value Ratio.

**Supplementary Table 6.** Regression models’ parameters for the association with verbal memory scores when controlling for visual memory scores for Braak II SUVR.

| Rey Auditory Verbal Learning Test (verbal memory z-scores) | | | |
| --- | --- | --- | --- |
| Coefficients | Beta (95% CI) | T-value | *P* value |
| R Braak II SUVR | -0.08 (-0.21-0.05) | -1.223 | 0.223 |
| [^18^F]AZD4694 SUVR, mean (SD) | -0.21 (-0.34- -0.08) | -3.158 | **0.002** |
| Grey matter volume (residuals) | 0.14 (0.04-0.24) | 2.864 | **0.005** |
| AFLT DR z-score | 0.51 (0.4-0.63) | 8.736 | **<0.001** |
| Years of education | 0.09 (-0.01-0.19) | 1.874 | 0.063 |
| Age | -0.06 (-0.16-0.04) | -1.234 | 0.219 |
| Handedness (left) | 0.09 (-0.22-0.4) | 0.576 | 0.566 |
| Sex (female) | 0.36 (0.16-0.55) | 3.527 | **<0.001** |
| APOE4 status (positive) | -0.07 (-0.28-0.15) | -0.606 | 0.545 |
| Rey Auditory Verbal Learning Test (verbal memory z-scores) | | | |
| Coefficients | Beta (95% CI) | T-value | *P* value |
| L Braak II SUVR | -0.11 (-0.25-0.02) | -1.679 | 0.095 |
| [^18^F]AZD4694 SUVR, mean (SD) | -0.19 (-0.32- -0.06) | -2.797 | **0.006** |
| Grey matter volume (residuals) | 0.15 (0.05-0.25) | 2.933 | **0.004** |
| AFLT DR z-scores | 0.51 (0.4-0.63) | 8.872 | **<0.001** |
| Years of education | 0.1 (-0.01-0.19) | 1.951 | 0.053 |
| Age | -0.06 (-0.16-0.03) | -1.282 | 0.202 |
| Handedness (left) | 0.07 (-0.24-0.38) | 0.448 | 0.655 |
| Sex (female) | 0.36 (0.16-0.56) | 3.61 | **<0.001** |
| APOE4 status (positive) | -0.05 (-0.27-0.17) | -0.434 | 0.665 |

Standardized β coefficients for predictor and covariates. Abbreviations: AFLT DR, Aggie Figures Learning Test Delayed Recall; L, left; R, right; SD, Standard Deviation; SUVR, Standardized Uptake Value Ratio.

**Supplementary Table 7.** Regression models’ parameters for the association with visual memory scores when controlling for verbal memory scores for Braak II SUVR

| Aggie Figures Learning Test (visual/nonverbal memory z-scores) | | | |
| --- | --- | --- | --- |
| Coefficients | Beta (95% CI) | T-value | *P* value |
| R Braak II SUVR | -0.14 (-0.28-0.01) | -1.924 | 0.056 |
| [^18^F]AZD4694 SUVR, mean (SD) | -0.07 (-0.21-0.08) | -0.922 | 0.358 |
| Grey matter volume | 0.03 (-0.08-0.14) | 0.546 | 0.586 |
| RAVLT DR z-scores | 0.61 (0.48-0.75) | 8.736 | **<0.001** |
| Years of education | 0.05 (-0.06-0.16) | 0.939 | 0.349 |
| Age | -0.03 (-0.14-0.08) | -0.506 | 0.614 |
| Handedness (left) | -0.17 (-0.5-0.17) | -0.969 | 0.334 |
| Sex (female) | -0.23 (-0.45- -0.01) | -2.015 | **0.046** |
| APOE4 status (positive) | 0.04 (-0.19-0.28) | 0.367 | 0.714 |
| Aggie Figures Learning Test (visual/nonverbal memory z-scores) | | | |
| Coefficients | Beta (95% CI) | T-value | *P* value |
| L Braak II SUVR | -0.08 (-0.23-0.07) | -1.028 | 0.306 |
| [^18^F]AZD4694 SUVR, mean (SD) | -0.1 (-0.25-0.05) | -1.299 | 0.196 |
| Grey matter volume | 0.03 (-0.08-0.14) | 0.568 | 0.571 |
| RAVLT DR z-scores | 0.63 (0.49-0.77) | 8.872 | **<0.001** |
| Years of education | 0.05 (-0.06-0.16) | 0.883 | 0.379 |
| Age | -0.02 (-0.13-0.09) | -0.393 | 0.695 |
| Handedness (left) | -0.18 (-0.52-0.16) | -1.027 | 0.301 |
| Sex (female) | -0.24 (-0.47- -0.01) | -2.105 | **0.037** |
| APOE4 status (positive) | 0.03 (-0.22-0.27) | 0.208 | 0.835 |

Standardized β coefficients for predictor and covariates. Abbreviations: L, left; R, right; RAVLT DR, Rey Auditory Verbal Learning Test Delayed Recall; SD, Standard Deviation; SUVR, Standardized Uptake Value Ratio.

**Supplementary Table 8.** Regression models’ parameters for the association with verbal memory scores when controlling for visual memory scores for Braak III SUVR.

| Rey Auditory Verbal Learning Test (verbal memory z-scores) | | | |
| --- | --- | --- | --- |
| Coefficients | Beta (95% CI) | T-value | *P* value |
| R Braak III SUVR | -0.1 (-0.22-0.03) | -1.569 | 0.119 |
| [^18^F]AZD4694 SUVR, mean (SD) | -0.22 (-0.34- -0.1) | -3.728 | **<0.001** |
| Grey matter volume (residuals) | 0.13 (0.03-0.23) | 2.537 | **0.012** |
| AFLT DR z-score | 0.5 (0.38-0.62) | 8.173 | **<0.001** |
| Years of education | 0.09 (-0.01-0.19) | 1.891 | 0.06 |
| Age | -0.08 (-0.18-0.02) | -1.509 | 0.133 |
| Handedness (left) | 0.07 (-0.24-0.38) | 0.416 | 0.678 |
| Sex (female) | 0.36 (0.16-0.55) | 3.547 | **<0.001** |
| APOE4 status (positive) | -0.08 (-0.29-0.13) | -0.717 | 0.474 |
| Rey Auditory Verbal Learning Test (verbal memory z-scores) | | | |
| Coefficients | Beta (95% CI) | T-value | *P* value |
| L Braak III SUVR | -0.14 (-0.26- -0.01) | -2.205 | **0.029** |
| [^18^F]AZD4694 SUVR, mean (SD) | -0.2 (-0.32- -0.09) | -3.419 | **<0.001** |
| Grey matter volume (residuals) | 0.12 (0.02-0.22) | 2.441 | **0.016** |
| AFLT DR z-scores | 0.49 (0.37-0.61) | 8.317 | **<0.001** |
| Years of education | 0.1 (0.01-0.2) | 2.013 | **0.046** |
| Age | -0.08 (-0.19-0.02) | -1.649 | 0.101 |
| Handedness (left) | 0.05 (-0.26-0.36) | 0.315 | 0.753 |
| Sex (female) | 0.35 (0.16-0.55) | 3.57 | **<0.001** |
| APOE4 status (positive) | -0.07 (-0.28-0.14) | -0.673 | 0.502 |

Standardized β coefficients for predictor and covariates. Abbreviations: AFLT DR, Aggie Figures Learning Test Delayed Recall; L, left; R, right; SD, Standard Deviation; SUVR, Standardized Uptake Value Ratio.

**Supplementary Table 9.** Regression models’ parameters for the association with visual memory scores when controlling for verbal memory scores for Braak III SUVR

| Aggie Figures Learning Test (visual/nonverbal memory z-scores) | | | |
| --- | --- | --- | --- |
| Coefficients | Beta (95% CI) | T-value | *P* value |
| R Braak III SUVR | -0.21 (-0.34- -0.07) | -3.09 | **0.002** |
| [^18^F]AZD4694 SUVR, mean (SD) | -0.08 (-0.21-0.05) | -1.187 | 0.237 |
| Grey matter volume | 0.01 (-0.11-0.11) | 0.026 | 0.979 |
| RAVLT DR z-scores | 0.58 (0.44-0.72) | 8.173 | **<0.001** |
| Years of education | 0.05 (-0.05-0.16) | 1.01 | 0.314 |
| Age | -0.07 (-0.18-0.05) | -1.16 | 0.248 |
| Handedness (left) | -0.21 (-0.54-0.12) | -1.256 | 0.211 |
| Sex (female) | -0.21 (-0.43-0.01) | -1.877 | 0.062 |
| APOE4 status (positive) | 0.03 (-0.2-0.26) | 0.281 | 0.779 |
| Aggie Figures Learning Test (visual/nonverbal memory z-scores) | | | |
| Coefficients | Beta (95% CI) | T-value | *P* value |
| L Braak III SUVR | -0.14 (-0.27- -0.01) | -2.039 | **0.043** |
| [^18^F]AZD4694 SUVR, mean (SD) | -0.1 (-0.23-0.04) | -1.412 | 0.16 |
| Grey matter volume | 0.01 (-0.1-0.12) | 0.202 | 0.84 |
| RAVLT DR z-scores | 0.6 (0.46-0.74) | 8.317 | **<0.001** |
| Years of education | 0.05 (-0.05-0.16) | 1.006 | 0.316 |
| Age | -0.05 (-0.16-0.07) | -0.818 | 0.414 |
| Handedness (left) | -0.2 (-0.54-0.14) | -1.179 | 0.24 |
| Sex (female) | -0.23 (-0.45- -0.01) | -2.054 | **0.042** |
| APOE4 status (positive) | 0.02 (-0.22-0.25) | 0.14 | 0.889 |

Standardized β coefficients for predictor and covariates. Abbreviations: L, left; R, right; RAVLT DR, Rey Auditory Verbal Learning Test Delayed Recall; SD, Standard Deviation; SUVR, Standardized Uptake Value Ratio.

**Supplementary Table 10.** Regression models’ parameters for the association with verbal memory scores when controlling for visual memory scores for Braak IV SUVR.

| Rey Auditory Verbal Learning Test (verbal memory z-scores) | | | |
| --- | --- | --- | --- |
| Coefficients | Beta (95% CI) | T-value | *P* value |
| R Braak IV SUVR | -0.07 (-0.2-0.05) | -1.166 | 0.245 |
| [^18^F]AZD4694 SUVR, mean (SD) | -0.23 (-0.34- -0.11) | -3.764 | **<0.001** |
| Grey matter volume (residuals) | 0.13 (0.03-0.23) | 2.574 | **0.011** |
| AFLT DR z-score | 0.51 (0.39-0.63) | 8.353 | **<0.001** |
| Years of education | 0.09 (-0.01-0.19) | 1.82 | 0.071 |
| Age | -0.07 (-0.17-0.03) | -1.382 | 0.169 |
| Handedness (left) | 0.09 (-0.22-0.4) | 0.562 | 0.575 |
| Sex (female) | 0.35 (0.15-0.55) | 3.499 | **<0.001** |
| APOE4 status (positive) | -0.09 (-0.3-0.12) | -0.831 | 0.407 |
| Rey Auditory Verbal Learning Test (verbal memory z-scores) | | | |
| Coefficients | Beta (95% CI) | T-value | *P* value |
| L Braak IV SUVR | -0.09 (-0.22- -0.03) | -1.502 | 0.135 |
| [^18^F]AZD4694 SUVR, mean (SD) | -0.21 (-0.33- -0.09) | -3.511 | **<0.001** |
| Grey matter volume (residuals) | 0.13 (0.03-0.23) | 2.569 | **0.011** |
| AFLT DR z-scores | 0.51 (0.39-0.62) | 8.556 | **<0.001** |
| Years of education | 0.09 (-0.01-0.19) | 1.854 | 0.066 |
| Age | -0.08 (-0.18-0.03) | -1.481 | 0.141 |
| Handedness (left) | 0.08 (-0.23-0.39) | 0.53 | 0.597 |
| Sex (female) | 0.36 (0.16-0.56) | 3.567 | **<0.001** |
| APOE4 status (positive) | -0.08 (-0.29-0.13) | -0.748 | 0.456 |

Standardized β coefficients for predictor and covariates. Abbreviations: AFLT DR, Aggie Figures Learning Test Delayed Recall; L, left; R, right; SD, Standard Deviation; SUVR, Standardized Uptake Value Ratio.

**Supplementary Table 11.** Regression models’ parameters for the association with visual memory scores when controlling for verbal memory scores for Braak IV SUVR

| Aggie Figures Learning Test (visual/nonverbal memory z-scores) | | | |
| --- | --- | --- | --- |
| Coefficients | Beta (95% CI) | T-value | *P* value |
| R Braak IV SUVR | -0.21 (-0.34- -0.08) | -3.133 | **0.002** |
| [^18^F]AZD4694 SUVR, mean (SD) | -0.07 (-0.2-0.06) | -1.043 | 0.3 |
| Grey matter volume | 0.01 (-0.11-0.11) | -0.045 | 0.964 |
| RAVLT DR z-scores | 0.58 (0.45-0.72) | 8.353 | **<0.001** |
| Years of education | 0.05 (-0.06-0.15) | 0.898 | 0.37 |
| Age | -0.06 (-0.17-0.05) | -1.144 | 0.255 |
| Handedness (left) | -0.17 (-0.5-0.16) | -1.014 | 0.312 |
| Sex (female) | -0.21 (-0.43-0.01) | -1.913 | 0.058 |
| APOE4 status (positive) | 0.01 (-0.21-0.24) | 0.123 | 0.902 |
| Aggie Figures Learning Test (visual/nonverbal memory z-scores) | | | |
| Coefficients | Beta (95% CI) | T-value | *P* value |
| L Braak IV SUVR | -0.15 (-0.28-0.01) | -2.152 | **0.033** |
| [^18^F]AZD4694 SUVR, mean (SD) | -0.08 (-0.22-0.05) | -1.235 | 0.219 |
| Grey matter volume | 0.01 (-0.1-0.12) | 0.189 | 0.85 |
| RAVLT DR z-scores | 0.61 (0.47-0.74) | 8.556 | **<0.001** |
| Years of education | 0.05 (-0.06-0.16) | 0.893 | 0.373 |
| Age | -0.05 (-0.16-0.06) | -0.881 | 0.38 |
| Handedness (left) | -0.17 (-0.51-0.16) | -1.024 | 0.307 |
| Sex (female) | -0.22 (-0.44-0.01) | -1.958 | 0.052 |
| APOE4 status (positive) | 0.02 (-0.21-0.25) | 0.15 | 0.881 |

Standardized β coefficients for predictor and covariates. Abbreviations: L, left; R, right; RAVLT DR, Rey Auditory Verbal Learning Test Delayed Recall; SD, Standard Deviation; SUVR, Standardized Uptake Value Ratio.

**Supplementary Table 12.** Regression models’ parameters for the association with verbal memory scores when controlling for visual memory scores for Braak V SUVR.

| Rey Auditory Verbal Learning Test (verbal memory z-scores) | | | |
| --- | --- | --- | --- |
| Coefficients | Beta (95% CI) | T-value | *P* value |
| R Braak V SUVR | -0.05 (-0.16-0.07) | -0.748 | 0.455 |
| [^18^F]AZD4694 SUVR, mean (SD) | -0.24 (-0.35- -0.12) | -4.033 | **<0.001** |
| Grey matter volume (residuals) | 0.13 (0.03-0.24) | 2.596 | **0.01** |
| AFLT DR z-score | 0.52 (0.4-0.64) | 8.668 | **<0.001** |
| Years of education | 0.09 (-0.01-0.19) | 1.808 | 0.072 |
| Age | -0.07 (-0.17-0.04) | -1.279 | 0.203 |
| Handedness (left) | 0.09 (-0.22-0.4) | 0.566 | 0.572 |
| Sex (female) | 0.35 (0.15-0.55) | 3.47 | **<0.001** |
| APOE4 status (positive) | -0.09 (-0.31-0.12) | -0.885 | 0.378 |
| Rey Auditory Verbal Learning Test (verbal memory z-scores) | | | |
| Coefficients | Beta (95% CI) | T-value | *P* value |
| L Braak V SUVR | -0.05 (-0.17- -0.06) | -0.91 | 0.364 |
| [^18^F]AZD4694 SUVR, mean (SD) | -0.23 (-0.35- -0.12) | -3.926 | **<0.001** |
| Grey matter volume (residuals) | 0.13 (0.03-0.24) | 2.611 | **0.01** |
| AFLT DR z-scores | 0.52 (0.4-0.63) | 8.834 | **<0.001** |
| Years of education | 0.09 (-0.01-0.19) | 1.834 | 0.069 |
| Age | -0.07 (-0.17-0.03) | -1.328 | 0.186 |
| Handedness (left) | 0.09 (-0.22-0.4) | 0.562 | 0.575 |
| Sex (female) | 0.35 (0.15-0.55) | 3.496 | **<0.001** |
| APOE4 status (positive) | -0.09 (-0.31-0.12) | -0.879 | 0.381 |

Standardized β coefficients for predictor and covariates. Abbreviations: AFLT DR, Aggie Figures Learning Test Delayed Recall; L, left; R, right; SD, Standard Deviation; SUVR, Standardized Uptake Value Ratio.

**Supplementary Table 13.** Regression models’ parameters for the association with visual memory scores when controlling for verbal memory scores for Braak V SUVR

| Aggie Figures Learning Test (visual/nonverbal memory z-scores) | | | |
| --- | --- | --- | --- |
| Coefficients | Beta (95% CI) | T-value | *P* value |
| R Braak V SUVR | -0.18 (-0.3- -0.05) | -2.751 | **0.007** |
| [^18^F]AZD4694 SUVR, mean (SD) | -0.09 (-0.22-0.04) | -1.341 | 0.182 |
| Grey matter volume | -0.01 (-0.12-0.11) | -0.105 | 0.916 |
| RAVLT DR z-scores | 0.6 (0.47-0.74) | 8.668 | **<0.001** |
| Years of education | 0.05 (-0.06-0.16) | 0.909 | 0.365 |
| Age | -0.07 (-0.18-0.05) | -1.156 | 0.249 |
| Handedness (left) | -0.18 (-0.51-0.16) | -1.045 | 0.298 |
| Sex (female) | -0.21 (-0.43-0.01) | -1.921 | 0.056 |
| APOE4 status (positive) | 0.01 (-0.23-0.23) | 0.011 | 0.991 |
| Aggie Figures Learning Test (visual/nonverbal memory z-scores) | | | |
| Coefficients | Beta (95% CI) | T-value | *P* value |
| L Braak V SUVR | -0.12 (-0.25-0.01) | -1.957 | 0.052 |
| [^18^F]AZD4694 SUVR, mean (SD) | -0.1 (-0.23-0.03) | -1.486 | 0.139 |
| Grey matter volume | 0.01 (-0.11-0.12) | 0.135 | 0.893 |
| RAVLT DR z-scores | 0.62 (0.48-0.76) | 8.834 | **<0.001** |
| Years of education | 0.05 (-0.06-0.16) | 0.911 | 0.364 |
| Age | -0.05 (-0.17-0.06) | .0.885 | 0.377 |
| Handedness (left) | -0.17 (-0.51-0.16) | -1.011 | 0.313 |
| Sex (female) | -0.23 (-0.45- -0.01) | -1.994 | **0.048** |
| APOE4 status (positive) | -0.01 (-0.23-0.23) | -0.007 | 0.995 |

Standardized β coefficients for predictor and covariates. Abbreviations: L, left; R, right; RAVLT DR, Rey Auditory Verbal Learning Test Delayed Recall; SD, Standard Deviation; SUVR, Standardized Uptake Value Ratio.

**Supplementary Table 14.** Regression models’ parameters for the association with verbal memory scores when controlling for visual memory scores for Braak VI SUVR.

| Rey Auditory Verbal Learning Test (verbal memory z-scores) | | | |
| --- | --- | --- | --- |
| Coefficients | Beta (95% CI) | T-value | *P* value |
| R Braak VI SUVR | -0.06 (-0.17-0.06) | -0.981 | 0.328 |
| [^18^F]AZD4694 SUVR, mean (SD) | -0.23 (-0.35- -0.12) | -3.946 | **<0.001** |
| Grey matter volume (residuals) | 0.13 (0.03-0.23) | 2.501 | **0.013** |
| AFLT DR z-score | 0.52 (0.41-0.63) | 8.951 | **<0.001** |
| Years of education | 0.09 (-0.01-0.19) | 1.860 | 0.065 |
| Age | -0.07 (-0.17-0.03) | -1.309 | 0.192 |
| Handedness (left) | 0.07 (-0.24-0.39) | 0.473 | 0.637 |
| Sex (female) | 0.35 (0.15-0.55) | 3.5 | **<0.001** |
| APOE4 status (positive) | -0.1 (-0.31-0.11) | -0.955 | 0.341 |
| Rey Auditory Verbal Learning Test (verbal memory z-scores) | | | |
| Coefficients | Beta (95% CI) | T-value | *P* value |
| L Braak VI SUVR | -0.07 (-0.18- -0.04) | -1.201 | 0.232 |
| [^18^F]AZD4694 SUVR, mean (SD) | -0.23 (-0.34- -0.11) | -3.863 | **<0.001** |
| Grey matter volume (residuals) | 0.13 (0.03-0.23) | 2.521 | **0.013** |
| AFLT DR z-scores | 0.52 (0.41-0.64) | 9.124 | **<0.001** |
| Years of education | 0.09 (-0.01-0.19) | 1.873 | 0.063 |
| Age | -0.07 (-0.17-0.03) | -1.356 | 0.177 |
| Handedness (left) | 0.07 (-0.24-0.38) | 0.444 | 0.658 |
| Sex (female) | 0.36 (0.16-0.56) | 3.532 | **<0.001** |
| APOE4 status (positive) | -0.1 (-0.32-0.11) | -0.975 | 0.331 |

Standardized β coefficients for predictor and covariates. Abbreviations: AFLT DR, Aggie Figures Learning Test Delayed Recall; L, left; R, right; SD, Standard Deviation; SUVR, Standardized Uptake Value Ratio.

**Supplementary Table 15.** Regression models’ parameters for the association with visual memory scores when controlling for verbal memory scores for Braak VI SUVR

| Aggie Figures Learning Test (visual/nonverbal memory z-scores) | | | |
| --- | --- | --- | --- |
| Coefficients | Beta (95% CI) | T-value | *P* value |
| R Braak VI SUVR | -0.09 (-0.21- -0.03) | -1.427 | 0.155 |
| [^18^F]AZD4694 SUVR, mean (SD) | -0.11 (-0.25-0.02) | -1.676 | 0.096 |
| Grey matter volume | 0.01 (-0.11-0.12) | 0.145 | 0.885 |
| RAVLT DR z-scores | 0.63 (0.49-0.76) | 8.951 | **<0.001** |
| Years of education | 0.05 (-0.06-0.16) | 0.907 | 0.366 |
| Age | -0.04 (-0.15-0.08) | -0.629 | 0.53 |
| Handedness (left) | -0.19 (-0.53-0.15) | -1.109 | 0.269 |
| Sex (female) | -0.24 (-0.46- -0.01) | -2.088 | **0.038** |
| APOE4 status (positive) | -0.02 (-0.25-0.22) | -0.135 | 0.893 |
| Aggie Figures Learning Test (visual/nonverbal memory z-scores) | | | |
| Coefficients | Beta (95% CI) | T-value | *P* value |
| L Braak VI SUVR | -0.03 (-0.15-0.09) | -0.46 | 0.646 |
| [^18^F]AZD4694 SUVR, mean (SD) | -0.13 (-0.26-0.01) | -1.928 | 0.056 |
| Grey matter volume | 0.02 (-0.09-0.14) | 0.401 | 0.689 |
| RAVLT DR z-scores | 0.64 (0.5-0.78) | 9.124 | **<0.001** |
| Years of education | 0.04 (-0.06-0.15) | 0.805 | 0.422 |
| Age | -0.02 (-0.13-0.09) | -0.376 | 0.708 |
| Handedness (left) | -0.17 (-0.52-0.17) | -0.993 | 0.322 |
| Sex (female) | -0.25 (-0.48- -0.03) | -2.212 | **0.028** |
| APOE4 status (positive) | -0.01 (-0.24-0.22) | -0.088 | 0.93 |

Standardized β coefficients for predictor and covariates. Abbreviations: L, left; R, right; RAVLT DR, Rey Auditory Verbal Learning Test Delayed Recall; SD, Standard Deviation; SUVR, Standardized Uptake Value Ratio.

**Supplementary Figure 2. The magnitude of right tau asymmetry is associated with worse visual memory scores.** Association between laterality index and A) visual delayed recall scores and B) verbal delayed recall scores in the whole sample. Negative index scores indicate right asymmetry, while positive index scores indicate left asymmetry. Scatterplots display the relationship between residuals of the laterality index and residuals of memory scores when correcting for global amyloid, grey matter volume, age, sex, years of education, APOE status and handedness.

**Supplementary Figure 3. The association between tau asymmetry and memory scores is not significant when segregating by cognitive status.** Association between laterality index and A) visual delayed recall scores and B) verbal delayed recall scores by cognitive status. Negative index scores indicate right asymmetry, while positive index scores indicate left asymmetry. Scatterplots display the relationship between residuals of the laterality index and residuals of memory scores when correcting for global amyloid, grey matter volume, age, sex, years of education, APOE status and handedness.

**Supplementary Figure 4. The magnitude of right tau asymmetry is associated with worse visual memory scores in participants with significant tau in any Braak region.** Association between laterality index and A) visual delayed recall scores and B) verbal delayed recall scores by significant tau in any PET-Braak area. Negative index scores indicate right asymmetry, while positive index scores indicate left asymmetry. Scatterplots display the relationship between residuals of the laterality index and residuals of memory scores when correcting for global amyloid, grey matter volume, age, sex, years of education, APOE status and handedness.

**Supplementary figure 5.** **The laterality index and visual memory are significantly and marginally associated, irrespective of APOE4 carriership.** The plot shows partial correlation coefficients. The associations are significant for APOE4 non-carriers, and marginal for APOE4 non-carriers. Once again, associations with verbal memory are nonexistent.

**Supplementary figure 6.** **Tau imbalance is slightly skewed to the right in our sample.** D’Agostino skewness test was performed in individuals that were positive in at least one of the PET-Braak stages and was significant.

**Supplementary figure 7.** **Tau load tends to lateralize in the temporal meta-ROI.** Absolute Laterality Index (LI) is associated with tau SUVR in the temporal meta-ROI. Laterality, irrespective of the side, is associated to higher tau SUVR values. Pearson correlation coefficient is shown. Participants are segregated by Braak Stage. Absolute LI index is calculated by turning all negative (right lateralization) values into positive values.

**Supplementary Figure 8. The association between tau load and visual memory scores continues to predominate in the right temporal lobe in right-handed individuals only.** Verbal (top) memory scores are also similarly related to tau loads bilaterally, although it seems that some regions on the left display strongest associations. T-statistical parametric maps were corrected for multiple comparisons using a random field theory cluster threshold of *p*<.001, overlaid on the Alzheimer's Disease Neuroimaging Initiative reference template. Two linear regression models were used, where either verbal delayed recall or visual delayed recall scores were entered as outcome variables and [^18^F]-MK6240 as predictor. Grey matter volume, age, sex, years of education, APOE status and amyloid-β positivity were used as covariates.

**Supplementary figure 9.** **The association between tau and recall scores is stronger than that between amyloid and recall scores when including individuals outside of the AD spectrum.** This larger sample featured an addition of 34 participants that were cognitively impaired but amyloid negative.

**Supplementary figure 10.** **The significant association between amyloid and recall scores disappears when using Braak I tau load.** This larger sample featured an addition of 34 participants that were cognitively impaired but amyloid negative.

**Supplementary figure 11.** **The associations between tau and memory in Braak I are similar across hemispheres.** The plot shows beta coefficients for the independent associations between predictors and visual or verbal memory scores.
